# Dielectric Properties in Oriented and Unoriented Membranes Based on Poly(Epichlorohydrin-co-Ethylene Oxide) Copolymers: Part III

**DOI:** 10.3390/polym14071369

**Published:** 2022-03-28

**Authors:** B. Pascual-Jose, Alireza Zare, Silvia De la Flor, José Antonio Reina, M. Giamberini, A. Ribes-Greus

**Affiliations:** 1 Institute of Technology of Materials (ITM), Universitat Politècnica de València (UPV), 46022 Camí de Vera, Spain; borpasjo@doctor.upv.es; 2 Department of Chemical Engineering (DEQ), Universitat Rovira I Virgili, Av. Païssos Catalans, 26, 43007 Tarragona, Spain; alireza.zare@urv.cat (A.Z.); marta.giamberini@urv.cat (M.G.); 3 Department of Mechanical Engineering, Universitat Rovira i Virgili (URV), Av. Països Catalans, 26, 43007 Tarragona, Spain; silvia.delaflor@urv.cat; 4 Department of Analytical Chemistry and Organic Chemistry, Universitat Rovira i Virgili (URV), C/Marcel.lí Domingo s/n, 43007 Tarragona, Spain; joseantonio.reina@urv.cat

**Keywords:** broadband dielectric spectroscopy, dielectric relaxation spectra, macromolecular cooperativity, segmental dynamics, poly(epichlorohydrin-co-ethylene oxide), electrical and protonic conductivity

## Abstract

The dielectric spectra and conductivity properties of neat poly(epichlorohydrin-co-ethylene oxide)(PECH-co-EO) copolymer and two modified copolymers with a 20% or 40% of dendron 3,4,5-tris[4-(*n*-dodecan-1-yloxy)benzyloxy] benzoate units were analysed. A process of thermal orientation was applied to the copolymers to fine-tune the molecular motion of the side chains and determine their validity for cation transport materials. The study was conducted using Dielectric Thermal Analysis (DETA). The spectra of the modified unoriented and oriented copolymers consisted of five dielectric relaxations (δ, γ, β, α_Tg_, and α_melting_). The analysis of the relaxations processes shows that as the grafting with the dendron units increases, both the lateral and main chains have a greater difficulty moving. The thermal orientation induces in the main chain partial crystallization, including the polyether segments, and modifies the cooperative motion of the main chain associated with the glass transition (α_Tg_). A deep analysis of the electrical loss modulus revealed that the degree of modification only modifies the temperature peak of each relaxation, and this effect is more perceived if the dendron unit content is higher (40%). The thermal orientation process seems equal to the spectra of CP20-O and CP40-O to the point that the degree of modification does not matter. Nevertheless, the fragility index denotes the differences in the molecular motion between both copolymers (40% and 20%) due to the thermal orientation. The study of the electric conductivity showed that the ideal long-range pathways were being altered by neither the thermal orientation process nor the addition of dendrimers. The analysis of the through-plane proton conductivity confirmed that the oriented copolymer with the highest concentration of dendrimers was the best performer and the most suitable copolymer for proton transport materials.

## 1. Introduction

Numerous works exist studying the cation transport phenomena [1,2,3,4,5,6,7]. Extensive research has been done to find new polymers that provide a good cation performance similar to or even better than Nafion, the benchmark material in proton conductivity [8,9,10,11,12,13,14,15].

Poly(epichlorohydrin) (PECH) grafted with dendritic side groups has been studied for these applications due to its availability to form channels, which facilitate the mobility of cations [16]. Percec et al. described the self-organized dendritic side groups and backbone. They demonstrated the final properties to selectively transport charges are dependent on the shape of the columnar self-assembly of the dendrimers, which design the supramolecular architecture [17,18,19]. Tylkowski et al. [20] prepared a series of copolymers of PECH with large supramolecular systems, which have a columnar structure that allowed the formation of ion channels on the inner part. Dendronized polymers and copolymers of poly(epichlorohydrin) (PECH) and poly[2-(aziridin-1-yl) ethanol] (PAZE), with different proportions of dendritic 3,4,5-tris[4-(*n*-dodecan-1-yloxy)benzyloxy]benzoate side groups, has been widely investigated by our research group [21,22,23,24,25,26,27,28,29].

These membranes, in which the polymer columns were homeotropically oriented, have been submitted to thermal treatment during membrane preparation and were analysed elsewhere by the same authors [25,30]. These copolymers show an ability to change their shape, achieve orientation, and slightly crystallize. This behaviour was deeply analysed by dielectric thermal analysis, supported by Differential Scanning Calorimetry (DSC), X-ray diffraction (XRD), and ^13^C Cross Polarization Magic Angle Spinning (CP-MAS) NMR, which allowed to characterize these materials as far as their structure and tendency to crystallize is concerned. These results indicate that modified PECHs were more flexible than PAZE copolymers and offered a higher free volume at a higher degree of modifications. Several factors, such as the number of dendritic side groups and their orientation, are responsible for cations mobility through channels.

Recently, the membranes prepared with dendronized poly (epichloroydrin-co-ethylene oxide), which present 20% and 40% of grafted tap dendrons, have been intensely studied in Part 1 and 2 of [31,32]. In these studies, PECH-co-EO units were chemically modified with 20% (CP20) and 40% (CP40) of tap dendrons. In Part 1, properties using differential scanning calorimetry (DSC), polarized optical microscopy (POM), X-ray diffraction (XRD), and dynamic mechanical thermal analysis (DMTA) were analysed and discussed. It was proven that all of the copolymers exhibited liquid crystalline columnar mesophase and partial main chain crystallinity. The orientation method produces an increment of the crystalline fractions and induces the self-assembly of the polymer chains into columnar structures. It allows understanding the evolution of the self-assembly process that occurs in these ionic channels and the final organization of the polymeric columns to optimize the design of membranes. In Part 2, technics, such as atomic force microscopy (AFM), field-emission scanning electron microscopy (FESEM), contact angle (CA) analysis, water uptake, proton conductivity, and linear sweep voltammetry (LSV), were carried out. Results obtained confirm that the presence of two different amounts of grafted dendrons affects oriented membranes’ transport capacity. It was concluded that the presence of water is not necessary to improve the cation transport in CP20 and CP40; only the presence of defined cationic channels is relevant. Besides, CP40 possesses channels with larger diameters and better-defined inner structures.

In this work, the same liquid crystalline poly(epichlorohydrin-co-ethylene oxide)(PECH-co-EO), grafted with 20% (CP20) and 40% (CP40) side chain dendron 3,4,5-tris[4-(*n*-dodecan-1-yloxy)benzyloxy] benzoate (Tap), were analysed by dielectric thermal analysis. The ethylene oxide unit in the polymeric backbone of P(ECH-co-EO) may act as a spacer, thus modifying the large supramolecular systems mobility because a large portion of free space is required to move self-organized dendritic side groups and backbone. In addition, it could favour interaction with cations and provide water-independent selective transport due to the electronegative oxygen atoms systems. Furthermore, the columns were submitted to an orientation process, which was of paramount importance to obtaining effective transport.

In this context, the characterization of the dielectric spectrum and the electric and protonic conductivity determines the backbone’s role in self-assembly and the interaction between the dendritic side groups. Therefore, a deep analysis of the dielectric spectrum and conductive properties, carried out in this work, can allow fine-tuning the copolymers’ design to ease the mobility of cations.

## 2. Materials and Methods

### 2.1. Materials and Membrane Preparation

Tetrahydrofuran (THF), P(ECH-co-EO) with PECH/PEO 1:1, and Tetrabutylammonium bromide (TBAB) ≥ 99% were supplied by either Fluka (Madrid, Spain) or Sigma-Aldrich (Madrid, Spain). The synthesis of the dendritic mesogenic groups and the copolymers was performed as described elsewhere [21]

The labelling of the copolymers was performed according to the degree of modification. Thus, the labels CP0, CP20, and CP40 correspond to the P(ECH-co-EO) samples with a degree of modification of 0% (neat), 20%, and 40%, respectively. Membranes were prepared by immersion precipitation method. The modified copolymer was dissolved in THF (30% *w*/*w*). After that, the homogeneous solution was cast by a casting machine (K-paint applicator, RK Paintcoat Instruments Ltd., Litlington, UK) on an FEP (Fluorinated Ethylene Propylene) sheet support with a controlled thickness (gap size 300 μm). Then, the support, including the wet film on top, was immersed in a bath of Milli-Q water in which the polymeric membrane was formed with an asymmetric structure. After 24 h, the formed membrane was dried overnight at room temperature. Moreover, the membranes were vacuum dried at room temperature 48 h before weighing. In addition, the oriented samples of the CP20 and CP40 were prepared. The polymeric membrane (approx. 2 cm diameter) was placed on a hot stage (Linkam TP92, Linkam Scientific Instruments Ltd., Tadworth, UK) to achieve the homeotropically oriented structure of modified copolymer during the *baking* process, described as follows. For annealing, membranes were heated up to 140 °C. They were kept at the same temperature for 30 min. Then, they were slowly cooled (0.1 °C/min) to 107 °C, where they were held for 120 h. Afterwards, the membranes were allowed to cool to room temperature at 10 °C/min. Finally, the uniform membranes were left at room temperature for 1 h and then detached from FEP support. These samples were labelled as CP20-O and CP40-O, respectively.

### 2.2. Membrane Characterization

The dielectric thermal analysis (DETA) was performed using a Dielectric Spectrometer from Novocontrol Technologies GmbH & Co. KG, Hundsangen, Germany. The measurements were performed in the frequency range of 10^−1^ to 10^7^ Hz between 123 K to 383 K, under isothermal conditions by increasing steps of 10 K. The dielectric experiments were performed in a cell constituted by two gold electrodes, where the sample electrode assembly (SEA) was located.

The analysis was performed as explained elsewhere [33]. Briefly, the resultant dielectric spectra were fitted using the Havriliak–Negami (HN) functions [34,35] by adding as many HN functions as needed. All the characteristic parameters of each relaxation process were determined, as shown in Equation (1).

(1) $$\varepsilon^{*}\left(\omega \right)-\varepsilon_{\infty }=\underset{K}{\sum }Im\;\left[\frac{\Delta \varepsilon }{{\left(1+{\left(i\omega \tau_{HN}\right)}^{\alpha_{k}}\right)}^{\beta_{k}}}\right]$$

where *τ_HN_* is the Havriliak–Negami relaxation time. Thus, the sub-index *k* represents the number of the individual *HN* contributions; the parameters *a* and *b* correspond to the width and asymmetry broadening of the relaxation time distributions’ relaxation peak; Δ*ε* is the value of the dielectric intensity or relaxation strength.

**Figure 1:**
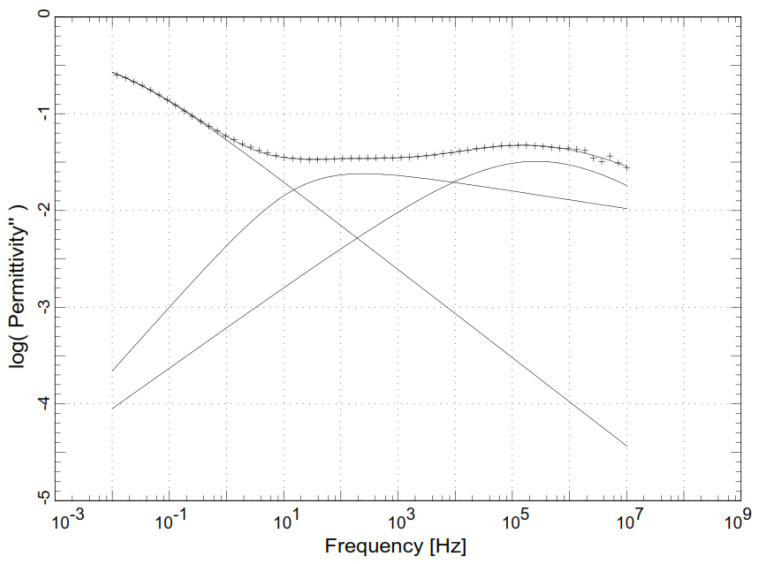
Example of HN fitting functions.

Thus, the isothermal dielectric loss modulus was fitted using at least three Havriliak–Negami functions, i.e., one for the main segmental relaxation and two or three for the secondary ones, as Figure 1 shows.

The Eyring model, as derived by Starkweather [36,37,38,39,40], was used to discriminate the macromolecular nature of the dielectric relaxations. Determining the cooperative or non-cooperative nature of the molecular motions is crucial to studying the relationships between the relaxation time (*τ*) and the temperature.

(2) $$E_{a}=RT\;\left[22.92+\ln T\right]$$

where *R* (J·mol·K^−1^) is the ideal gas constant, and *E_a_* (kJ · mol^−1^) refers to the apparent activation energy. Indeed, if the dielectric relaxation has a non-cooperative origin; thus, an Arrhenius-like model [41] would be used. On the contrary, if the molecular motion has a cooperative source, then the Vogel–Fulcher–Tamman–Hesse (VFTH) model [42,43,44] is used so that the apparent activation energy (*E_a_*) and the fragility index (*m*) are determined.

(3) $$f_{max}=f_{0}\;\exp\left(\frac{-E_{a}}{RT}\right)$$

(4) $$\tau \left(T\right)=\tau_{0}\exp\left(\frac{B}{T-T_{VFTH}}\right)$$

(5) $$m=\frac{B\;T_{VFTH}}{\ln\left(10\right){\left(T_{g}-T_{VFTH}\right)}^{2}}$$

where *T_g_* (K) refers to the glass transition temperature, *T_VFTH_* (K) refers to the temperature obtained in the fit, *B* (K) is an activation parameter, *f*_0_ is a pre-exponential term (Hz), and *τ*_0_ is a pre-exponential term (s).

Moreover, the contributions of the dc and ac conductivities are assessed through Jonscher’s power law [45]; the proton conductivity, however, is determined employing the protonic resistance (*Ω*) and geometric factors (*L* refers to the thickness, and *A* refers to the cross sectional area) of the samples [41,46].

(6) $$\sigma \;\left(\omega \right)=\sigma_{DC}+\sigma_{AC}\left(\omega \right)=\sigma_{DC}+A\omega^{n}$$

(7) $$\sigma_{prot}=L/AR_{0}$$

## 3. Results

The previous results [30,44,45] indicate that dendrimer substitution and the thermal treatment applied during the preparation of the copolymers could slightly modify the molecular mobility and define the final properties of these materials. Consequently, deep characterization of the dielectric relaxation spectrum may clarify the role of the ethylene oxide unit in the polymeric backbone of poly (epichlorohydrin). The other two factors, dendrimer substitution and the thermal treatment, may increase chain flexibility, leading to a more straightforward arrangement and regular structure. Thus, the dielectric measurements were performed on CP0, 0% (neat), CP20, and CP40 copolymers corresponding to the P(ECH-co-EO) samples with a degree of modification of 20% and 40%, respectively. These membranes were also submitted to thermal treatment (CP20-O, CP40-O). The dielectric relaxation spectra were assessed in terms of the isochronal and isothermal curves of the copolymers.

### 3.1. Analysis of the Dielectric Spectrum of CP0

**Figure 2:**
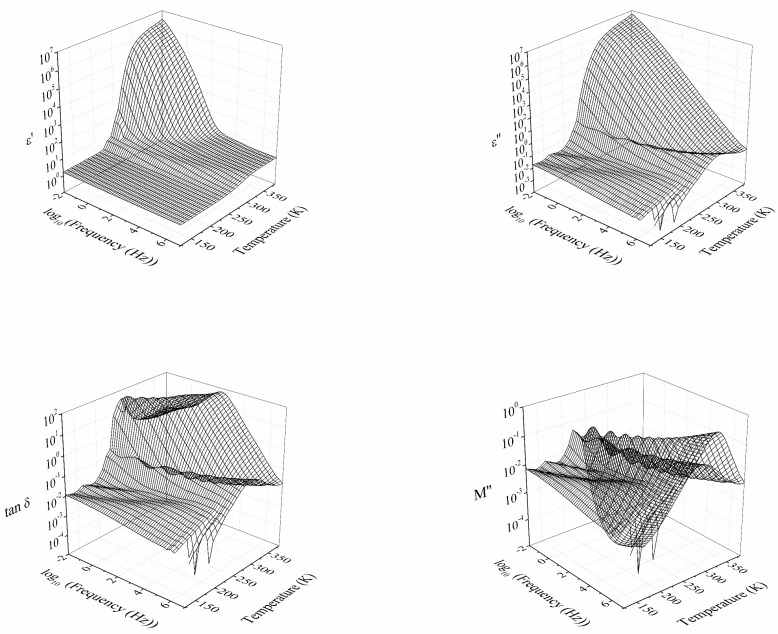
Three-dimensional plot of the dielectric spectra of the CP0 in terms of the real part of the dielectric permittivity (*ε*′), the imaginary part of the dielectric permittivity (*ε*″), the loss tangent (tan *δ*), and the imaginary part of the complex electric modulus (*M*″).

In Figure 2, the isothermal and isochronal 3D curves in terms of the real (*ε*′) and imaginary (*ε*″) part of the dielectric permittivity, the loss tangent (tan *δ*), and the electrical modulus are plotted for the unoriented copolymer. Similar curves were obtained for the thermally oriented copolymer. They were not included in this plot because the most significant differences will be more easily observed in the subsequent figures. In general terms, a complex dielectric relaxation spectrum was observed. Two or three relaxations were displayed at low temperatures that may be caused by different molecular motions related to the dendritic side groups. A remarkable feature of the dielectric spectrum of PEO is the tendency to merge several processes, and it is common to refer to them as β + γ′ or β + γ′ + γ [47]. The main segmental relaxation related to the glass transition appears at high temperatures. All relaxation processes are related to the chain motions in the amorphous or interface domain.

The γ relaxation occurs at low temperatures, between 123 K and 153 K, at the frequency of 10^−1^ Hz. This relaxation has been found to appear in the same temperature range as the γ relaxation of neat PEO. Some discrepancies have been raised about the origin of this molecular motion. This relaxation could be generated by local twisting in amorphous segments in the fold structure on crystal surfaces. Nonetheless, this dielectric relaxation could also be attributed to local motions in the amorphous phase, as it occurs in other crystalline polymers [47].

**Figure 3:**
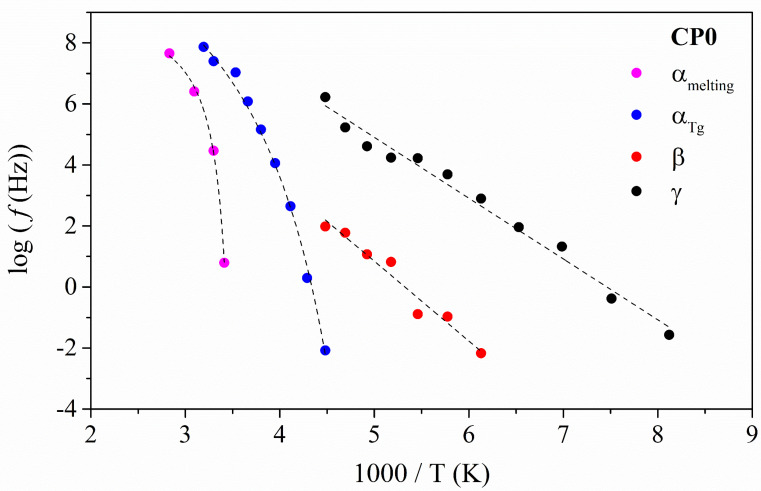
Arrhenius map for the neat copolymer (CP0). Dashed lines represent the line of best fit.

The β relaxation occurs between 163 K to 203 K at a frequency of 10^−1^ Hz. This molecular relaxation zone may be attributed to the sum of two dielectric relaxations of the neat PEO, the β and the γ′ relaxations, respectively. For this copolymer, both molecular relaxations at high frequencies have shown a tendency to merge. The γ′ relaxation is attributed to the motion of PEO segments in interface regions [47]. The β relaxation is attributed to cooperative motions in the amorphous part of PEO [47,48]. This point will be further discussed when analysing the Arrhenius map displayed in Figure 3.

The dielectric relaxation attributed to the glass transition of the PECH segments is found between 213 K and 313 K at a frequency of 10^−1^ Hz. This peak is also observed in the dielectric spectra of neat PECH, which may be associated with the glass transition of PECH. It tends to be the prominent relaxation, dominating the entire spectrum in this temperature range [48].

The higher temperature peak is observed between 323 K and 383 K at a frequency of 10^−1^ Hz. This dielectric relaxation is attributed to the molecular motion that promotes the melting.

The thermal dependence of the relaxation times (*τ*) with the temperature is plotted in Figure 3 to clarify the molecular origin of each one of these relaxations. The relaxations at low temperatures follow an Arrhenius-like model, thus indicating that their origin is related to non-cooperative molecular motions. The results are gathered in Table 1.

The apparent activation energies are gathered in Table 1. The values obtained are around 38 kJ·mol^−1^ for the γ relaxation and about 50 kJ·mol^−1^ for the β process. Some researchers [47] found lower values for the apparent activation energy (*E_a_*) of the γ′ relaxation (for 21 kJ·mol^−1^) and higher values for the β + γ′ process, around 188 kJ·mol^−1^. These differences may be due to the difficulty in separating each relaxation. In this work, the deconvolution process was carefully performed. The high Ea value, compared to a pure γ′ relaxation, indicates that a certain degree of cooperativity is involved in the β process, which reinforces the proposed assignation.

This difficulty is displayed in Figure 3. It is essential to consider because the interaction of the PECH segments with the PEO segments is why a complex relaxation zone in the amorphous domains is generated. In this figure, one can observe a mixture of both β and γ′ relaxations but neither the complete β nor the γ′ since the glass transition of the PECH segments is hampering them. Therefore, considering the values displayed in Table 1 and the above mentioned, it can be concluded that the frequency and temperatures assigned to the γ and the β are in accordance with those already published. Table 1 presents the best-fitted data.

On the other hand, the relaxation at high temperature shows that thermal dependence of the relaxation times is fitted to a Vogel–Fulcher–Tamman–Hesse (VFTH) model. This result confirms the cooperative nature of the molecular motions related to the glass transition. The best-fit parameters are gathered in Table 2.

The temperature–frequency range of the α_Tg_ coincides with the intervals provided by other authors for the glass transition of neat PECH [48,49]. The analysis of the fragility (*D*) denotes a somewhat strong character since systems are considered strong when values equal to or higher than 15 are obtained, meaning that this molecular motion shows a specific resistance to sudden changes in temperature. Thus, CP0 maintains its properties well at the temperature range when this relaxation is active, and it is confirmed by the low value displayed by the dilatation coefficient (*α*), whereas the free volume parameter (*Ø*) has a typical value for amorphous systems [50].

Close to glass transition relaxation, α_melting_ relaxation appears. This assignment is based on the temperature peak of this relaxation and the values displayed by the *m*, *α,* and *Ø* parameters. The fragility is very low, indicating a fragile behaviour (*D* ≤ 6) meaning high variability in the thermal properties of the CP0 in that temperature range. Again, this is validated by the high values displayed by the dilatation coefficient and the free volume parameters that a melting process can only explain.

### 3.2. Analysis of the Dielectric Spectra of the Modified Oriented and Unoriented Copolymers (CP40, CP40-O, CP20-O, and CP40-O)

**Figure 4:**
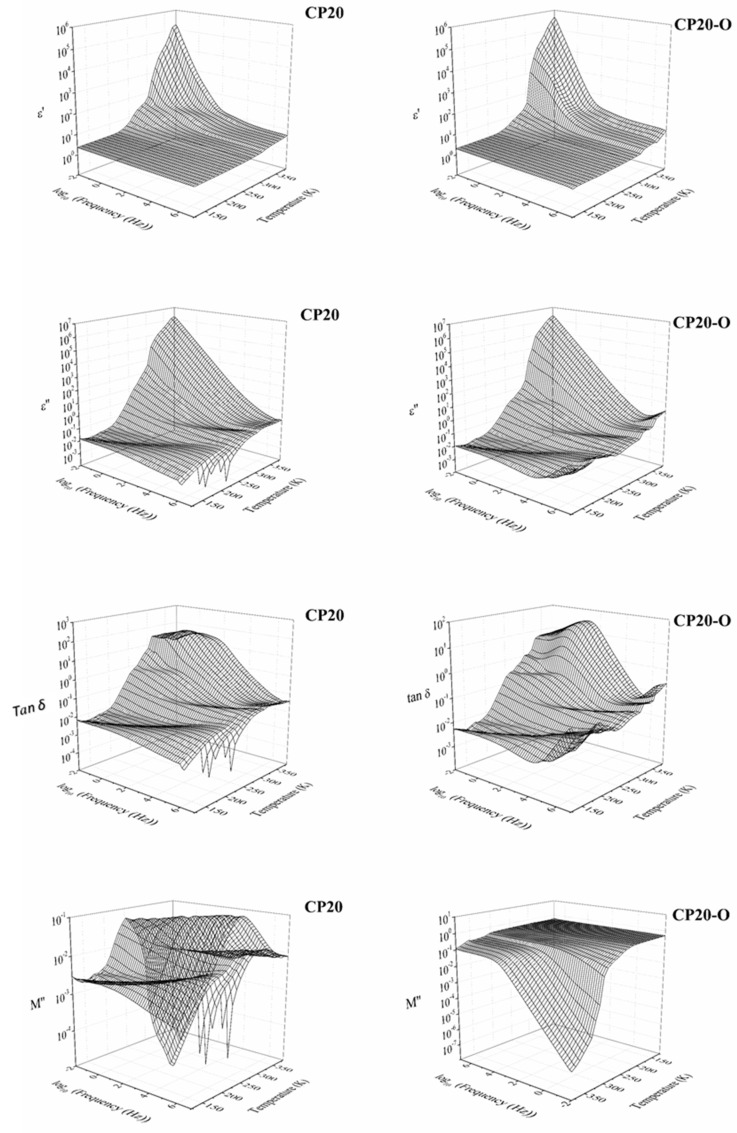
Three-dimensional plot of the dielectric spectra of the CP20 and CP20-O in terms of the real part of the dielectric permittivity (*ε*′), the imaginary part of the dielectric permittivity (*ε*″), the loss tangent (tan *δ*), and the imaginary part of the complex electric modulus (*M*″).

**Figure 5:**
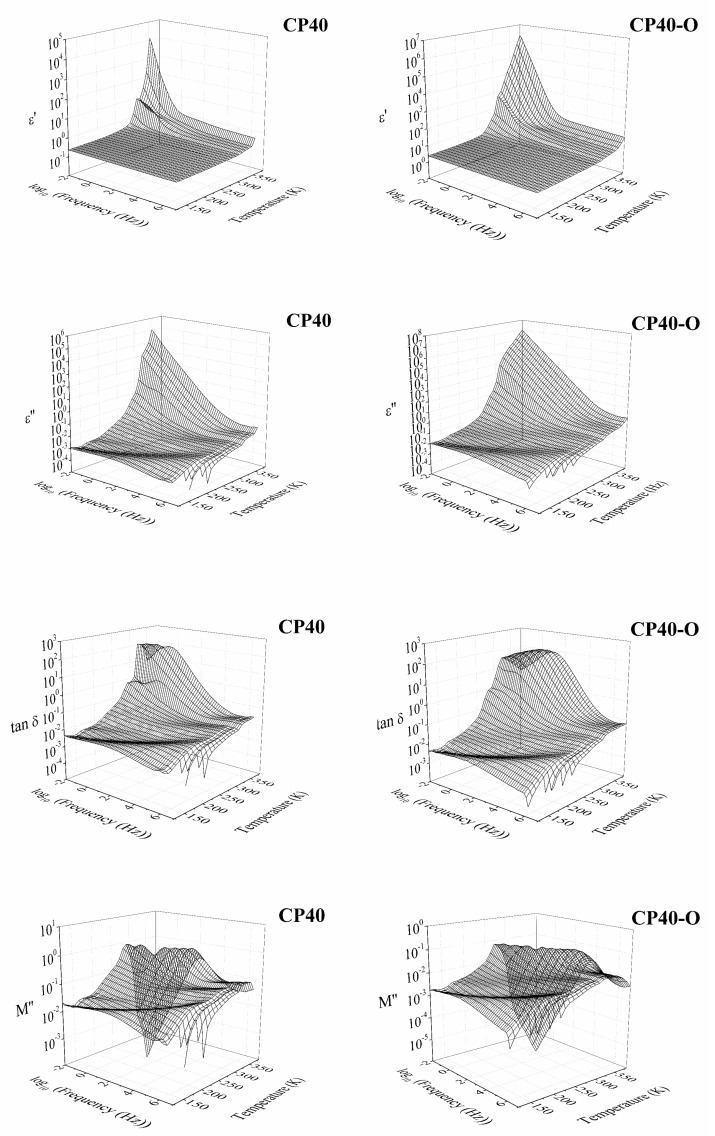
Three-dimensional plot of the dielectric spectra of the CP40 and CP40-O in terms of the real part of the dielectric permittivity (*ε*′), the imaginary part of the dielectric permittivity (*ε*″), the loss tangent (tan *δ*), and the imaginary part of the complex electric modulus (*M*″).

In Figure 4 and Figure 5, the isothermal and isochronal 3D curves in terms of the real (*ε*′) and imaginary (*ε*″) part of the dielectric permittivity, the loss tangent (tan *δ*), and the electrical modulus of unoriented and oriented CP20 and CP40 copolymers are plotted. In general terms, the dielectric spectra of CP20, CP20-O, CP40-O, and CP40-O copolymers also exhibit a complex spectrum with broad dielectric relaxation zones, which consists of five molecular motions: three at low temperatures (δ, γ, and β) and two at high temperatures (α_Tg_ and α_melting_).

**Figure 6:**
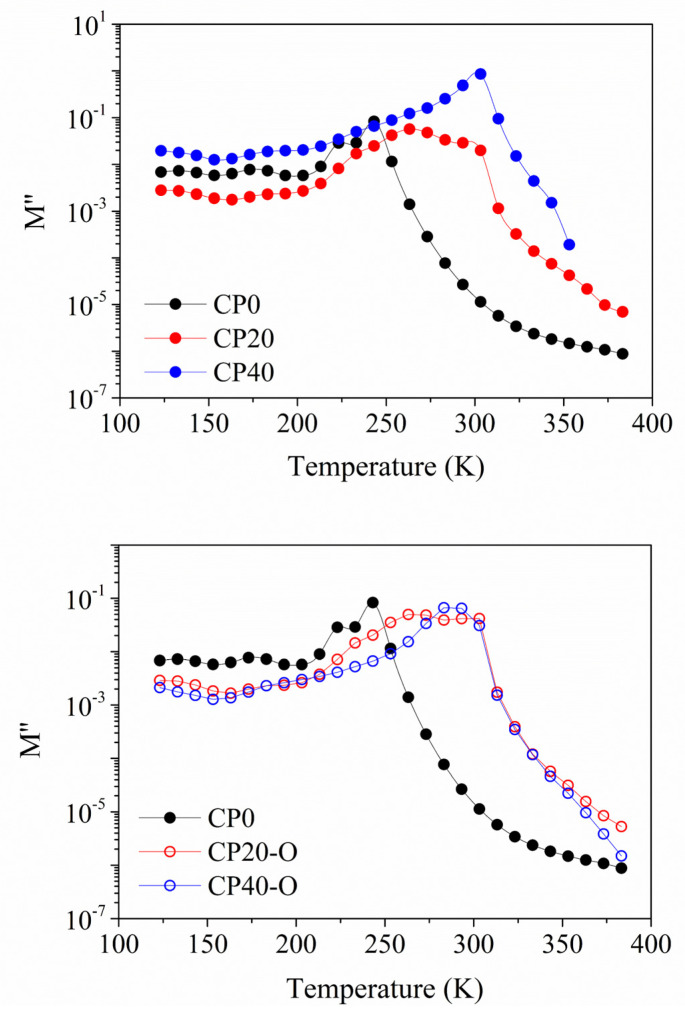
Isochronal curves for the imaginary part of the complex modulus (M*) at a frequency of 10^−1^ Hz (**Top**) for the neat and unoriented membranes; (**Bottom**) for the neat and oriented membranes.

A previous study [48] found that CP40 has a higher degree of crystallinity than CP20. To understand the differences between the increment in the degree of modification and the degree of crystallinity, electrical modulus, at the frequency of 1 Hz, are chosen because the *ε*′ and *ε*″ curves display no significant differences between the CP0 and CP20 membranes. In Figure 6, isochronal curves of electrical modulus are shown for a frequency of 10^−1^ Hz. Several dielectric relaxations are observed during the entire temperature range.

Changing the degree of modification from 20% to 40% does not impact the number of dielectric relaxations occurring in the spectra. However, the temperature range, where some of these processes occur, is shifted. However, it is interesting to note that the orientation process seems to equal the spectra of the CP20-O and CP40-O to the point that the degree of modification does not matter. This could mean that the same number of molecules are in motion in both cases (CP40 and CP40-O), but the difference would be in the level of restrictions found.

**Figure 7:**
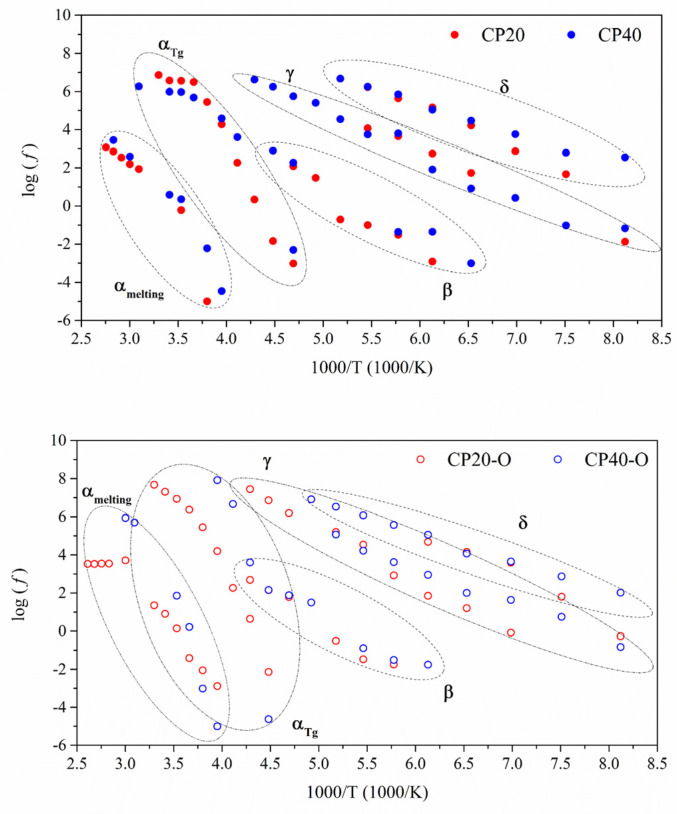
Arrhenius map for the unoriented (full symbols) and oriented (hollow symbols) modified copolymers.

**Figure 8:**
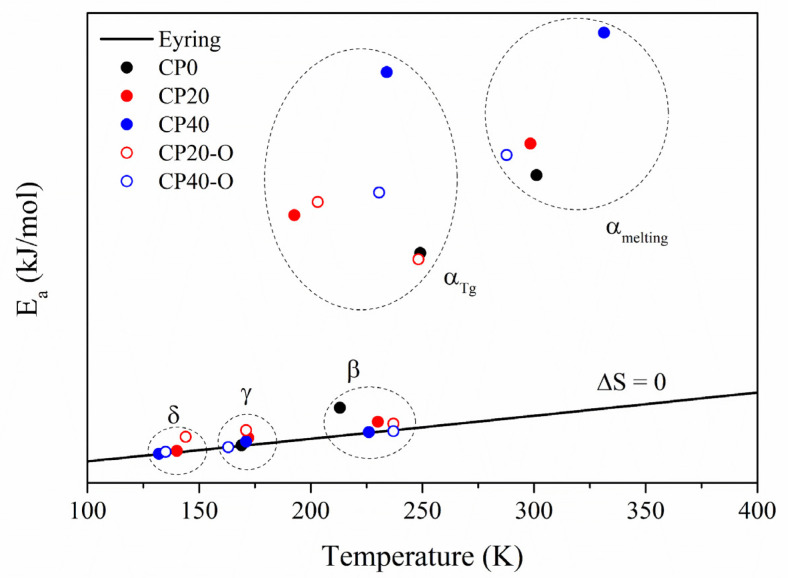
Eyring graph for all the dielectric relaxations of the neat and modified copolymers.

The thermal dependence of the relaxation times (*τ*) with the temperature is plotted in Figure 7 to clarify the molecular origin of each of these relaxations. It was also assessed through the Eyring model as derived by Stalkweather [36,37,38,39,40] in Figure 8. According to the Eyring model, the apparent activation energy (*E_a_*) is determined and compared to an equivalent calculated considering that Δ*S* = 0. Thus, if obtained values of *E_a_* are close to the zero-entropy line, these relaxations can be regarded as of intramolecular (or non-cooperative) origin since for this type of molecular relaxations, the contribution of the entropy can be disregarded. However, suppose the values are far away from the zero-entropy line. In that case, therefore, the dielectric relaxations are classified as of intermolecular (or cooperative) origin since its departure from the zero-entropy value indicates that entropy is playing a significant role. Thus, only cooperative motions would create a similar response.

As shown in Figure 8, the dielectric relaxations with a non-cooperative origin are the three located at low temperatures (δ, γ, and β) since their values are close to the zero-entropy line. On the other hand, the molecular motions located at high temperatures (α_Tg_ and α_melting_) are cooperative since their values lie far from the zero-entropy ones.

#### 3.2.1. Analysis of the Low-Temperature Relaxation Zone

**Figure 9:**
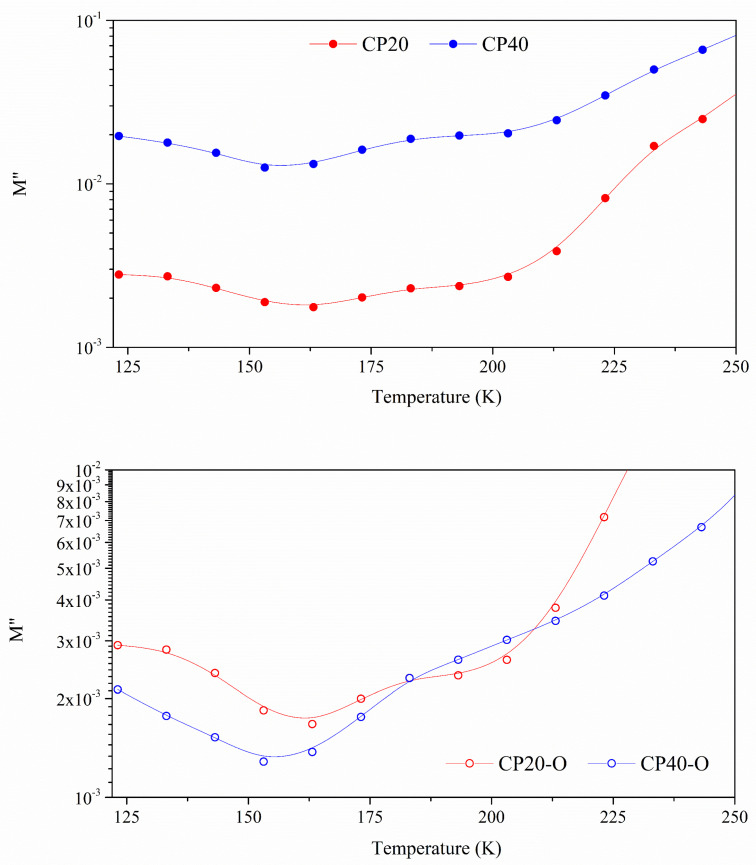
Loss modulus for the unoriented (**top**) and oriented (**bottom**) modified copolymers at 10^−1^ Hz frequency.

In Figure 9, the loss modulus is plotted for the unoriented and oriented membranes from 123 K to 250 K at the frequency of 10^−1^ Hz. In this temperature range, three dielectric relaxations have already been labelled as δ, γ, and β. The δ and γ relaxations occur between 123 K and 173 K, and at this frequency, they are overlapped, hence the broadness of the peak.

According to Figure 7 and Figure 8, the δ relaxation occurs at lower temperatures than the γ relaxation of the CP0. From the Arrhenius map plots in Figure 7, concerning the δ relaxation, the orientation process does not seem to produce notable differences. The apparent activation energy (*E_a_*) values, gathered in Table 3, vary between 28–49 kJ/mol, according to the values found in previous work for similar PECH and PAZE copolymers [25,30]. As in these copolymers, the thermal orientation increases (*E_a_*) in both copolymers. Therefore, this dielectric relaxation is attributed to an intramolecular movement related to the benzyloxy group of the dendrimers and is labelled as δ relaxation. The organized structure hinders the motion of dendrimers, increasing the energy barrier. On the other hand, the slightly higher values of the imaginary part of the electrical modulus achieved by CP40 to CP20 are logical since the more substituted copolymer has a higher dendrimers concentration. In both cases, thermal orientation decreases the values of the electrical modulus, especially in the CP40-O copolymer.

On the contrary, the γ relaxation coincides with the molecular arrangements observed in the neat copolymer. Therefore, this dielectric relaxation is attributed to the same movement that originates the γ relaxation in the CP0. Table 3 shows *E_a_* values that vary between 36–56 kJ/mol and are in accordance with the value of 37 kJ/mol found for CP0. Since the origin of this relaxation is due to the PEO segments, the concentration of dendrimers should not play a significant role in its dynamics. Instead, the degree of crystallization seems to be the main factor to explain the different values obtained [31]. While CP20 and CP40 display a degree of crystallization around 30%, the difference is more significant for oriented membranes. The high level of ordering of the main chain at CP40 in combination with the thermal orientation process seems to favour the dynamics of the γ relaxation.

The β relaxation is observed from 173 K to 213 K at 10^−1^ Hz. It has the exact macromolecular origin that in CP0, i.e., the motion of the amorphous regions of the PEO segments. This is a very complex zone, as displayed by Figure 9. It can be observed how the signal of the β relaxation is shallow and is immediately overlapped by the α relaxation, which is related to the glass transition of PECH, and it tends to prevail [51]. It is more evident for the modified oriented membranes (CP20-O, CP40-O). The Arrhenius map shows a tendency of all the modified copolymers to shift towards lower frequencies although the temperature range is maintained. Consequently, there is a lowering of the *E_a_* values found to vary between 52–65 kJ/mol compared to the value of 80 kJ/mol displayed by the CP0, as Table 3 displays. In this case, it is interesting to note that the process of thermal orientation cannot be considered the root cause since the variation found in the value of *E_a_* between the unoriented and its equivalent oriented membrane is negligible. Similar *E_a_* values were found for CP40-O and the CP40. However, the significant differences between the degree of crystallization would also indicate that the degree of crystallinity should not be considered responsible for this behaviour [31]. Another option affecting *E_a_* values could be the different concentrations of dendrimers; nevertheless, they are attached to the PECH segments, thus meaning that the percentage of PEO remains the same for all the samples [31]. Therefore, the reason for the shift in frequencies and the consequent lowering in apparent activation energy must be related to confinement effects [46].

#### 3.2.2. Analysis of the High-Temperature Relaxation Zone

**Figure 10:**
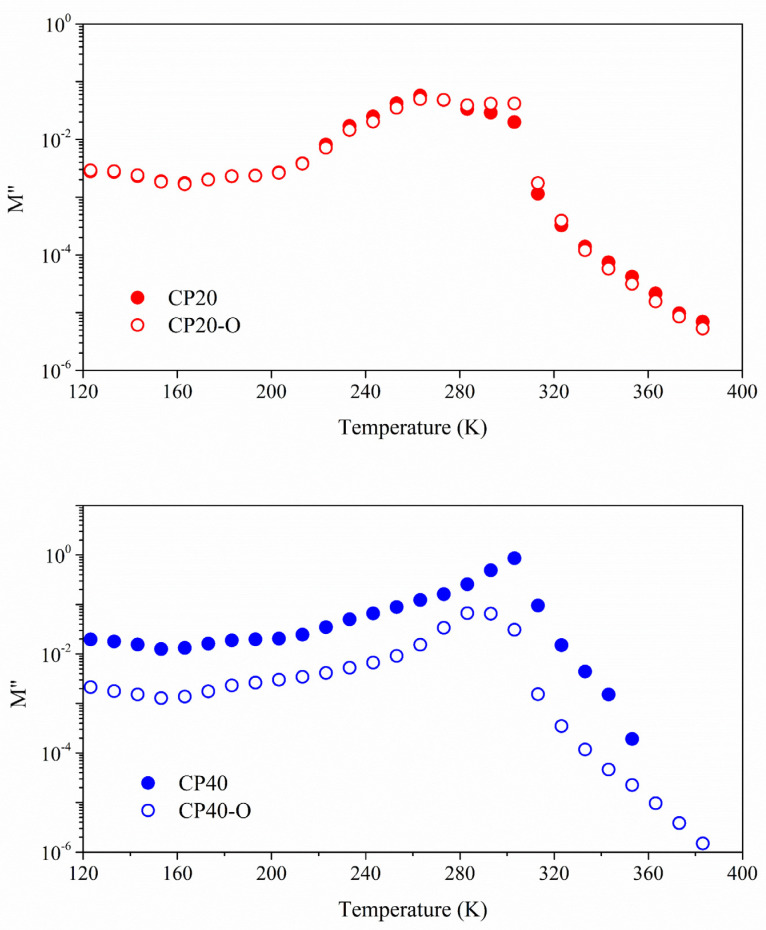
Loss modulus for the modified oriented and unoriented copolymers at 10^−1^ Hz frequency.

In Figure 10, the loss modulus is plotted for the unoriented and oriented membranes. Two dielectric relaxations are found at the high-temperature range that has already been labelled as α_Tg_ and α_Tmelting_. The former is attributed to the glass transition of the PECH, and the second corresponds to motions of both the initial segment of the melting and the end segment of the clearing transition. The thermal orientation process is not producing any significant variation in the dielectric spectra of the CP20. On the contrary, notable differences are observed in the case of the CP40, where the CP40-O displays lower values of the loss modulus than the CP40. The analysis of the thermal dependence of the relaxation times will provide further details on the molecular structure and, more specifically, the changes produced by the orientation process.

Either the glass transition of the PECH or the motions giving rise to the melting and clearing processes are cooperative motions. Therefore, a Vogel–Fulcher–Tamman–Hesse model is needed to determine the thermal dependence of the relaxation times. The parameters for the best fit are gathered in Table 4 and Table 5 for the α_Tg_ and α_Tmelting_, respectively.

As the fragility index (m) of the CP0 is 55, adding the dendrimers tends to reduce the fragile behaviour in both copolymers CP20 and CP40, and the higher the concentration, the stronger it becomes. However, for the oriented copolymers, the values tend to increase the fragility index towards the CP0, especially in the case of the CP20, when the concentration of dendrimers is still low.

Concerning the free volume (Φ), the CP0 presented a value of 0.03, and the addition of a low concentration of dendrimers (CP20) does not produce changes in the free volume in both oriented and unoriented copolymers. These values agree with the values displayed for most of the polymeric systems, which lie in the 0.025 ± 0.005 range [51]. On the contrary, when the concentration of dendrimers increases (CP40), the free volume diminishes significantly. It is important to remark that the orientation process does not produce any variation in the available free volume. These results confirm that molecular motions are hindered in the case of the CP40, as is to be expected.

The *E_a_*_Tg_ of CP0 copolymer is 244.70 kJ/mol. The addition of dendrimers increases the apparent activation energy, which is higher in increasing the concentration of the dendrimers. The restrictions imposed by the higher concentration of dendrimers, resulting in less free space, are reflected by higher apparent activation energy and agree with the increment in the glass transition temperature found in the previous work [31]. Nevertheless, the orientation slightly increases the activation energy of CP20 and significantly decreases the value of the CP40. This result could mean that in CP40, the orientation of the dendrons favours the stabilization of the overall structure of the copolymer.

Regarding the α_melting_ relaxation, the derived VFTH parameters are gathered in Table 5. In general, the fragile behaviour prevails, and these copolymers are not thermally stable at this range of temperatures, which confirms previously collected DSC data [31]. The values displayed by the free volume parameter (Φ) in combination with the high values of the dilatation coefficient confirm the proposed origin of this molecular motion.

### 3.3. Analysis of the Electric and Proton Conductivity of the Neat and Modified Oriented and Unoriented Copolymers

It is necessary to maximize the protonic conductivity while minimizing the conduction of electrons to determine whether any of these membranes could work as a polymer electrolyte. The study of the charge transfer mechanism of the different copolymers will provide this information. Thus, it will be possible to find the ones that will perform appropriately.

#### 3.3.1. Electric Conductivity

**Figure 11:**
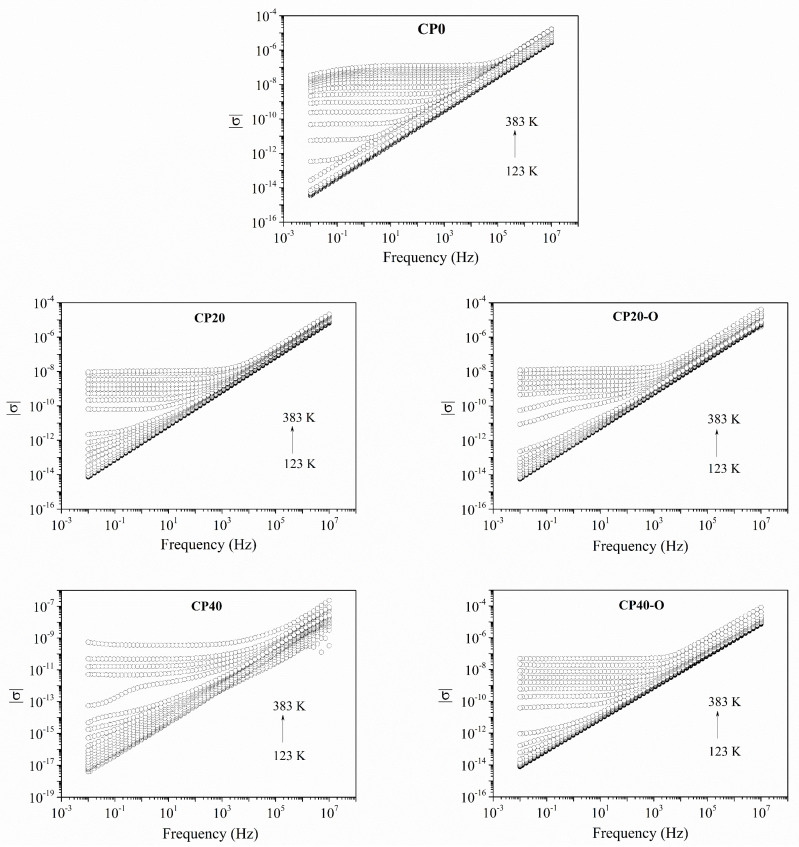
Modulus of the electric conductivity (|σ|) for the modified unoriented (CP20, CP40) and oriented (CP20-O, CP40-O) copolymers.

Figure 11 plots isochronal curves of the modulus of the electric conductivity (|σ|) for all the copolymers. The conductivity reaches a stable plateau at relatively low temperatures compared to the modified copolymers. Globally, the addition of the dendrimers slightly changed the conductivity, and both copolymers exhibit similar values. Nevertheless, it can be noted that at low concentrations, the CP20 and CP20-O behaviour is very similar and not far from the values of CP0. This result indicates that the charge transfer mechanisms have not been heavily modified yet. On the other hand, at higher dendrimer concentration, the hindering of the mobility by the restrictions imposed by the dendrimers disturb the charge transfer mechanisms and |σ| values decrease. It is noteworthy that the orientation process improves this situation and reduces the restrictions imposed by the dendrimers, obtaining better |σ| values than the unoriented copolymer comparable, if not better in some temperatures, to those of the CP0.

**Figure 12:**
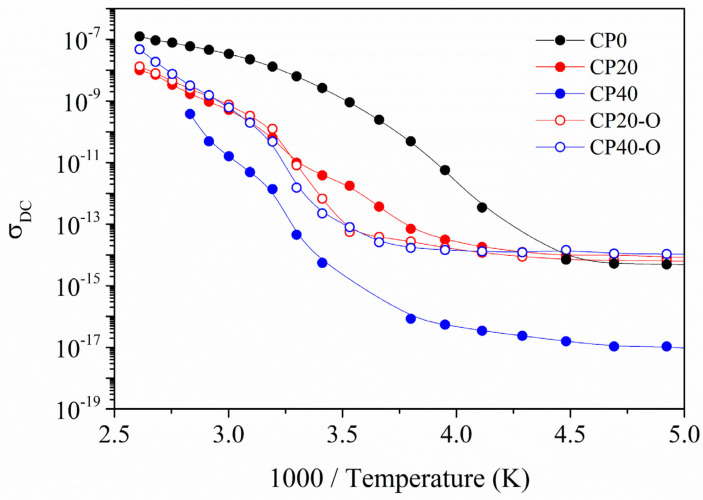
DC conductivity (σ_DC_) for the neat (CP0), modified unoriented (CP20, CP40), and oriented (CP20-O, CP40-O) copolymers.

Jonscher’s model is used to determine both components of the electric conductivity, i.e., the DC (σ_DC_) and the AC (σ_AC_), respectively. In Figure 12, the best fits for the modified and unmodified copolymers of σ_DC_ are plotted. In addition, the best fit for the Jonscher’s parameters are gathered in Table 6 for a selected range of temperatures. Regarding σ_DC_, as expected, the CP0 displays the highest values, whereas the CP20 and CP40 perform worse. However, these results confirm that the orientation of the main chain diminishes the restrictions imposed by the dendrimers and the values of conductivity, especially in CP40 copolymer obtained values of conductivity closer to the CP0.

Concerning the *A*-parameter, values are centred around 10^−13^ for all of the modified and unmodified copolymers except for the CP40. This is due to the low values of conductivity compared to the other copolymers, as already shown in Figure 11 and Figure 12. An important factor is the exponential parameter *n* because it gives an idea of the morphological texture. Accordingly, values nearer to 1 describe a system with ideal long-range pathways, while *n* values lower than 0.5 describe a conductive ion network with high levels of tortuosity [52]. Therefore, the results gathered in Table 6 show that all membranes display *n* values between 0.92 and 0.99. In addition, these results confirm that the thermal orientation process does not have a significant impact because it is not altering the values of the exponential parameter; there is only a smoothing of the values for all temperatures.

#### 3.3.2. Proton Conductivity

**Figure 13:**
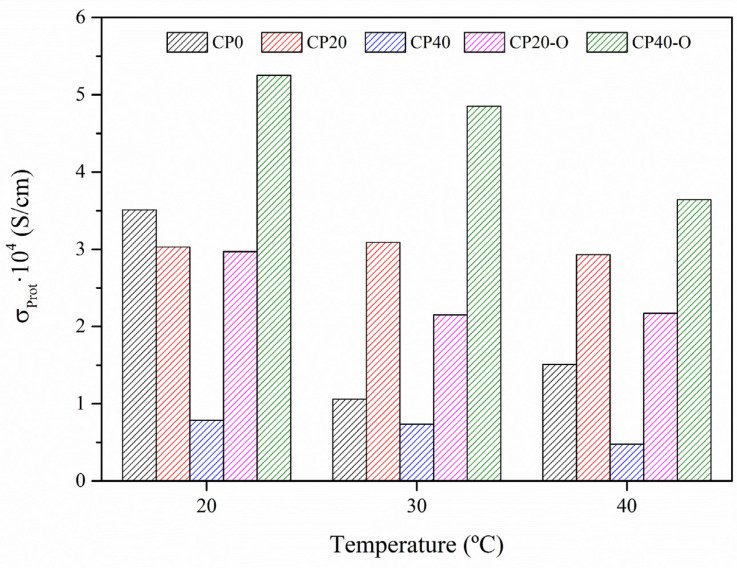
Through-plane proton conductivity (*σ_Prot_*) for the neat (CP0) and modified unoriented (CP20, CP40) and oriented (CP20-O, CP40-O) membranes at three different temperatures.

Figure 13 shows the values for the through-plane proton conductivity at three temperatures (293 K, 303 K, and 313 K) for all the copolymers.

At all these temperatures, the proton conductivity decreases as the concentrations of dendrimers increase. These results agree with our previous results, obtained in Parts I and II of this paper. The one-direction conductivity of cations depends on the effective cavity of the channel. The transport of cations in the narrow channels is responsible for limiting cations conductivity, and the polyether backbone also involves cation transport. However, in the case of protons, conduction also occurs through an additional coordination site, which lies on the lateral ester group that actively interacts with protons during their transport. It was observed that only protons could be transported via both the main chain and the lateral ester groups.

However, Figure 13 shows that the thermal orientation slightly decreases the proton conductivity at low dendrimer concentrations while increasing significantly at high dendrimers concentrations. The values obtained for CP40-O are higher than even CP0. These results confirm the significant impact of the dendron concentration and thermal orientation of the main chain in the design of these copolymers. Both factors alter the molecular motion, as confirmed by the dielectric spectrum and the charge transfer mechanism.

## 4. Conclusions

The spectra of the neat copolymer (CP0) consists of four dielectric relaxations (γ, β + γ′, α_Tg_, and α_melting_). Both poly(epichlorohydrin) and poly(ethylene oxide) display a similar glass transition temperature. However, poly(epichlorohydrin) prevails, generating a complex zone where the segmental and local motions of the amorphous fraction of poly(ethylene oxide) co-exist.

The spectra of the modified unoriented and oriented copolymers with 20% and 40% of dendrimers groups involves five dielectric relaxations (δ, γ, β, α_Tg_, and α_melting_). The δ relaxation was attributed to the local motions of the benzyloxy group of the dendrimers, and the orientation did not produce significant differences. The γ relaxation has the same molecular origin as CP0. A high degree of crystallinity in conjunction with the thermal orientation process appears to favour the dynamics of this dielectric relaxation. Regarding the β, the same complex dynamics between the cooperative and local motions of the poly(ethylene oxide) and the segmental motion of the poly(epichlorohydrin) are found. A shift in the temperature peaks in the modified unoriented and oriented copolymers with a logical decrease in the apparent activation energy was observed, related to confinement effects. The α_Tg_ is also ascribed to the cooperative motions of poly(epichlorohydrin). The analysis of the loss modulus revealed that the thermal orientation process did not produce any significant variation at low concentrations of dendrimers (CP20-O). On the contrary, significant variations were found at high concentrations of dendrimers (CP40-O). The α_melting_ displayed high values of both the free volume parameter and the dilatation coefficient, confirming its proposed macromolecular origin.

The study of the electric conductivity showed that the CP0 displayed the highest values of DC conductivity. In contrast, the CP40 was the worst performer due to the high levels of restrictions imposed by the high concentration of dendrimers. In addition, the exponent from Jonscher’s equation displayed values between 0.92 and 1 for all the copolymers studied, meaning that ideal long-range pathways were being altered by neither the thermal orientation process nor the addition of dendrimers.

The analysis of the through-plane proton conductivity confirms that the thermal orientation process combined with a high concentration of dendrimers (CP40-O) can enhance the proton conductivity while maintaining low enough electric conductivity values.

Finally, in the design of these copolymers to provide a proton conductivity similar to or even better than the benchmark materials, it is necessary to stand out the impact of both factors, the concentration of the dendrons groups, and thermal orientation of them and the main chain. Both together may improve the molecular motion and the charge transfer mechanism.

## Figures and Tables

**Table 1:** Apparent activation energy (*E_a_*) and temperature peak for CP0.

Relaxation	Slope	Intercept	*E_a_* (kJ·mol^−^^1^)	T_max_ (K) (1 kHz)	R^2^
γ	−1.99 ± 0.09	14.87 ± 0.53	38	183	0.981
β + γ′	−2.61 ± 0.23	13.92 ± 1.24	50	239	0.953

**Table 2:** Parameters for the VFTH model and derived parameters for studying the dynamic fragility of the neat copolymer (CP0).

Relaxation	Log *f*_0_	D	T_VFTH_ (K)	R^2^	Φ_Tg_	α_Tg_ × 10^4^ (K^−^^1^)	*E_a_*_Tg_ (kJ/mol)
α_Tg_	13.77 ± 0.56	10.32 ± 1.61	174	0.995	0.03	0.56	244.70
α_melting_	8.15 ± 0.13	0.58 ± 0.06	283	0.997	0.17	3.56	327.40

**Table 3:** The apparent activation energy (*E_a_*) and temperature peak for the modified oriented and unoriented copolymers.

Relaxation	Sample	Slope	Intercept	*E_a_* (kJ·mol^−1^)	T_max_ (K) (1 kHz)	R^2^
δ	CP20	−1.91 ± 0.19	16.58 ± 1.28	34	140	0.943
	CP40	−1.48 ± 0.11	14.23 ± 0.72	28	132	0.969
	CP20-O	−2.56 ± 0.31	20.85 ± 2.21	49	144	0.944
	CP40-O	−1.55 ± 0.05	14.48 ± 0.33	30	135	0.992
γ	CP20	−2.29 ± 0.06	16.70 ± 0.40	48	172	0.997
	CP40	−2.31 ± 0.18	16.54 ± 1.09	44	171	0.935
	CP20-O	−2.95 ± 0.23	20.33 ± 1.37	56	171	0.971
	CP40-O	−1.87 ± 0.08	14.52 ± 0.54	36	163	0.986
β	CP20	−3.38 ± 0.46	17.67 ± 2.47	65	230	0.914
	CP40	−2.83 ± 0.23	15.51 ± 1.29	54	226	0.974
	CP20-O	−3.29 ± 0.28	16.87 ± 1.40	63	237	0.965
	CP40-O	−2.71 ± 0.30	14.47 ± 1.58	52	237	0.942

**Table 4:** Parameters for the VFTH model and derived parameters for the study of the α_Tg_ relaxation of the modified oriented and unoriented copolymers.

Sample	Log *f*_0_	T_VFTH_ (K)	R^2^	m	Φ_Tg_	α × 10^4^ (K^−^^1^)	*E_a_*_Tg_ (kJ/mol)
CP20	15.41 ± 0.28	150.14 ± 23.34	0.963	25	0.03	0.36	285
CP20-O	9.19 ± 0.34	184.04 ± 1.16	0.998	59	0.03	0.61	299
CP40	8.25 ± 0.43	191.53 ± 3.18	0.994	12	0.01	0.19	437
CP40-O	15.23 ± 0.19	186.05 ± 0.86	0.998	22	0.01	0.24	309

**Table 5:** Parameters for the VFTH model and derived parameters for the study of the α_melting_ relaxation of the modified oriented and unoriented copolymers.

Sample	Log *f*_0_	T_VFTH_ (K)	R^2^	m	Φ_Tg_	α_Tg_ × 10^4^ (K^−^^1^)	*E_a_* (kJ/mol)
CP20	2.39 ± 0.45	238.07 ± 3.42	0.995	113	0.04	1.76	361
CP20-O	13.53 ± 0.87	178.59 ± 5.78	0.995	59	0.03	0.62	238
CP40	5.54 ± 0.46	218.17 ± 4.63	0.995	48	0.05	1.24	479
CP40-O	12.59 ± 0.68	206.21 ± 4.14	0.998	79	0.03	0.53	349

**Table 6:** Jonscher’s parameters for the modified oriented and unoriented copolymers.

Sample	Temperature (K)	σ_DC_	A	*n*	R^2^
CP20	293	3.86 × 10^−12^	4.60 × 10^−13^	0.94	0.999
	313	6.38 × 10^−11^	5.83 × 10^−13^	0.94	0.999
	323	2.17 × 10^−10^	5.98 × 10^−13^	0.95	0.999
	343	9.45 × 10^−10^	6.16 × 10^−13^	0.95	0.999
CP20-O	293	6.80 × 10^−13^	3.32 × 10^−13^	0.94	0.999
	313	1.25 × 10^−10^	6.75 × 10^−13^	0.93	0.999
	323	3.30 × 10^−10^	7.39 × 10^−13^	0.94	0.999
	343	1.42 × 10^−9^	7.53 × 10^−13^	0.95	0.999
CP40	293	5.55 × 10^−15^	1.32 × 10^−15^	0.94	0.991
	313	1.39 × 10^−12^	3.47 × 10^−15^	0.92	0.998
	323	4.89 × 10^−12^	2.43 × 10^−15^	0.96	0.999
	343	4.90 × 10^−11^	1.41 × 10^−15^	0.99	0.999
CP40-O	293	2.26 × 10^−13^	3.11 × 10^−13^	0.96	0.999
	313	4.82 × 10^−11^	5.17 × 10^−13^	0.94	0.999
	323	1.99 × 10^−10^	6.06 × 10^−13^	0.94	0.999
	343	1.55 × 10^−9^	7.95 × 10^−13^	0.94	0.999

## Data Availability

The data presented in this study are available on request from the corresponding author. The data are not publicly available due to it forms part of an ongoing study.
